# Hypercalcemia is common during *Pneumocystis* pneumonia in kidney transplant recipients

**DOI:** 10.1038/s41598-019-49036-w

**Published:** 2019-08-29

**Authors:** Aghilès Hamroun, Rémi Lenain, Linh Bui Nguyen, Paul Chamley, Séverine Loridant, Yann Neugebauer, Arnaud Lionet, Marie Frimat, Marc Hazzan

**Affiliations:** 1 University of Lille, Regional University Hospital Centre of Lille, Nephrology Dialysis and Kidney Transplantation Department, F-59000 Lille, France; 2 Nephrology Department, Beuvry Hospital, Bethune, France; 3 University of Lille, Regional University Hospital Centre of Lille, Microbiology Institute, Laboratory of Parasitology and Mycology, F-59000 Lille, France; 4 INSERM, UMR 995, F-59000 Lille, France; 5 Nephrology Department, Douai Hospital, Douai, France

**Keywords:** Fungal infection, Acid, base, fluid, electrolyte disorders

## Abstract

A few cases of hypercalcemia related to *Pneumocystis jirovecii* pneumonia (PJP) have previously been described, supposedly associated with an 1α-hydroxylase enzyme-dependent mechanism. The prevalence and significance of hypercalcemia in PJP remain unclear, especially in kidney transplant recipients (KTR) who frequently display hypercalcemia *via* persisting hyperparathyroidism. We here retrospectively identified all microbiologically-proven PJP in adult KTR from 2005 to 2017 in the Lille University Hospital, and studied the mineral and bone metabolism parameters during the peri-infectious period. Clinical features of PJP-patients were analyzed according to their serum calcium level. Hypercalcemia (12.6 ± 1.6 mg/dl) was observed in 37% (18/49) of PJP-patients and regressed concomitantly to specific anti-infectious treatment in all cases. No other cause of hypercalcemia was identified. In hypercalcemic patients, serum levels of 1,25-dihydroxyvitamin D were high at the time of PJP-diagnosis and decreased after anti-infectious treatment (124 ± 62 *versus* 28 ± 23 pg/mL, *p* = *0.006*) while PTH serum levels followed an inverse curve (35 ± 34 *versus* 137 ± 99 pg/mL, *p* = *0.009*), suggesting together a granuloma-mediated mechanism. Febrile dyspnea was less frequent in hypercalcemic PJP-patients compared to non-hypercalcemic (29 *versus* 67%). In summary, hypercalcemia seems common during PJP in KTR. Unexplained hypercalcemia could thus lead to specific investigations in this particular population, even in the absence of infectious or respiratory symptoms.

## Introduction

*Pneumocystis jirovecii* pneumonia (PJP) is an opportunistic infection that occurs in immunocompromised patients, such as solid organ transplant recipients^1^. Primary prophylactic treatment with trimethoprim-sulfamethoxazol (TMP-SMX) has significantly reduced the incidence of PJP, estimated nowadays between 0.3% and 2.6% of kidney transplant recipients (KTR)^2–4^. PJP remains a life-threatening infection with a mortality rate rising up to 30% in solid transplant recipients^5^, especially in the case of late diagnosis. Direct or molecular detection of *Pneumocystis jirovecii* in respiratory samples (bronchoalveolar lavage, BAL; sputum; oral washing; nasopharyngeal aspirate)^6,7^ in patients with respiratory symptoms (dyspnea, cough) generally leads to diagnosis. The clinical expression of PJP is however variable and may be insidious, making the diagnosis difficult.

Atypical cases of late onset and indolent PJP associated with hypercalcemia have been reported^8–20^. Increased calcium serum levels could be secondary to an extra-renal production of 1α-hydroxylase. Indeed, some reports of *Pneumocystis* pneumonia revealed the presence of inflammatory granulomas rich in macrophages and monocytes, capable of vitamin D activation and thereby inducing hypercalcemia^21,22^. Interestingly, 17 of the 21 described cases occurred in KTR, possibly suggesting a susceptibility in these patients for developing PJP-related hypercalcemia. Such observations are, however, sparse and the significance of this complication during PJP remains unclear, especially in KTR. Indeed, hypercalcemia is a frequent issue after kidney transplantation, persisting beyond the first year in 5 to 10% of recipients, mainly due to persistent hyperparathyroidism^23^.

In order to study the prevalence and potential mechanism(s) of hypercalcemia during PJP after kidney transplantation, we analyzed the parameters of phosphocalcic metabolism in a PJP-infected KTR cohort.

## Material and Methods

### Study population

All microbiologically-proven PJP cases in adult KTR were retrospectively collected from the laboratory database (Lille University Hospital, Institute of Microbiology) between January 2005 and August 2017. PJP diagnosis was defined according to the following criteria: 1- detection of *P. jirovecii* in BAL or oral washing specimen by direct microscopic examination (conventional stains -Giemsa and toluidine blue O-, indirect immunofluorescence -Monofluo kit *P. jirovecii*, Bio-Rad, Marnes- la-Coquette, France-), or *Pneumocystis*-specific quantitative real-time polymerase chain reaction assay (qPCR) targeting *P. jirovecii* mitochondrial large subunit (mtLSU) rRNA gene. All microbiological analyses were performed by the Laboratory of Parasitology and Mycology of Lille University Hospital; 2- a consistent clinical and/or radiological manifestation. Healthy carriers (asymptomatic or atypical presentation associated with a favorable outcome without PJP specific treatment) were excluded. All patients signed informed consent during the pre-transplant consultation, informing them of the potential use of their anonymized medical data for scientific purposes, unless they refused.

The study protocol has been certified to be in accordance with French laws by the Institutional Review Board of Regional University Hospital of Lille (Lille, France).

### Data collection and definitions

Patients’ features were collected from computer-based medical records: baseline characteristics at transplantation, immunosuppressive regimen, main comorbidities (pre-transplant diabetes, new onset diabetes after transplantation, chronic lung and/or heart diseases), and PJP episodes. Clinical symptoms of PJP upon admission were described as follows: febrile dyspnea, isolated fever, non-febrile respiratory symptoms (dyspnea and/or cough), or non-febrile alteration of general condition (isolated asthenia and/or anorexia). Simultaneous acute kidney injury (AKI) was defined according to the KDIGO-AKI classification (2012)^24^. Moreover, elements susceptible of interfering with the mineral and bone metabolism were carefully gathered, such as a history of parathyroidectomy, calcium or cholecalciferol supplementation, and use of thiazides or calcimimetics.

Biological data was collected from the laboratory database (Biochemistry laboratory, Lille University Hospital). Total calcium serum level was measured on a Roche Cobas 8000® module c701/702 (Basel, Switzerland) using colorimetric assay with o-cresolphthalein (CPC) method between 2005 and 2014, and then with NM-BAPTA method since 2015^25^. Hypercalcemia was defined above 10.5 mg/dL after adjustment to the albumin serum level (adjusted calcium = total calcium + (4.0 – albumin))^26^, according to the normal upper limit of the hospital laboratory.

As recommended in the international guidelines for KTR care, the ambulatory follow-up in our center is based on routine visits scheduled every 3 months^27^. To explore the mechanism of hypercalcemia, we collected specific biological parameters that are routinely realized in our department for kidney transplant follow-up (calcium, phosphorus, PTH, 25-hydoxyvitamin D, 1,25 dihydroxyvitamin D serum levels, urinary calcium-to-creatinine ratio), at the following time points: 1- at the annual transplantation consultation preceding PJP (3 to 12 months before PJP); 2- at the medical consultation preceding PJP (2 ± 1 months before PJP); 3- at the time of PJP-diagnosis; 4- at the end of PJP-treatment; 5- and finally, 4 ± 2 months after PJP resolution. Apart from biological examinations done during an emergency at the hospital admission for the PJP-episode, all the biological samples were taken in the same conditions (in the morning, after fasting). Kidney function (in the absence of AKI) was evaluated using an estimated glomerular filtration rate (eGFR) according to the 4-variable Modification of Diet in Renal Disease (MDRD) study equation.

### Statistical analysis

Quantitative variables were reported as mean ± standard deviation, or median [first-third quartiles], according to their distribution assessed by the Shapiro-Wilk test. The comparison of quantitative variables was performed by paired t-test or analysis of variance (when more than 2 groups), or Kruskall-Wallis test when appropriate. Categorical variables were expressed as an absolute number and percentage, and compared using a chi-square, Fisher exact test or Mac Nemar chi-square when appropriate. In the case of multiple comparisons, the adjusted Tukey or Benjamini & Hochberg post-tests were used for normal variables and non-parametric variables, respectively. Data was analyzed using the R program (R Core Team [2016]. R: A language and environment for statistical computing. R Foundation for Statistical Computing, Vienna, Austria; https://www.R-project.org/).

## Results

### Features of PJP-patients

*Pneumocystis jiroveci* was detected in 82 adult KTR (76 in BAL; 6 in oral washing specimens) from the microbiological database. *Pneumocystis*-specific qPCR was positive in all samples and, in 16 of them (19.5%), asci (former cysts) were also detected by optic microscopy. Thirty-three patients were considered as healthy carriers or false positive qPCR, none of them developing a PJP during a minimum 2-year follow-up in the absence of specific treatment.

PJP diagnosis was established in 49 of 2483 adult KTR (2%) between 2005 and 2017. The main features of the PJP patients are detailed in the Table 1. Briefly, these were predominantly middle-aged men (73.5%, 53.5 ± 15.3 years), in whom immunosuppression consisted in anti-thymoglobumin serum induction (61%) followed by a triple drug regimen including calcineurin inhibitors (CNI), steroids and mycophenolate mofetil (MMF). Twenty and 28.6% of patients were respectively treated for an acute rejection and/or an opportunistic infection (including Cytomegalovirus -CMV-, BK virus and Parvovirus B19 infection) in the year before PJP. All patients had PJP prophylaxis that consisted of TMP-SMX (400/80 mg) twice a week or pentamidine inhalations (300 mg monthly, in case of allergy to TMP-SMX) during the first 6 months after transplantation. The average eGFR at baseline (3 to 12 months before PJP) was 40.4 ± 17.1 ml/min/1.73 m^2^.

**Table 1:** Features of the PJP-population.

	***PJP***-***Patients*** (***n*** = ***49***)
Age (years)	53.5 ± 15.3
Male	36 (73.5%)
Mean interval KT-PJP (months), median [Q1; Q3]	13.4 [7.3; 54.4]
Baseline graft function (eGFR, MDRD, ml/min/1.73 m^2^), mean ± SD	40.4 ± 17.1
**Immunosuppression features**	
Retransplantation	10 (20.4%)
Induction treatment	
*Anti*-*thymocyte globulin*	30 (61.2%)
*CD25 monoclonal antibody*	19 (38.8%)
Immunosuppressive regimen	
*Steroid*	36 (77.6%)
*CNI*	46 (93.9%)
*MMF*	41 (83.7%)
*mTOR inhibitor*	8 (16.3%)
*Azathioprine*	1 (2.0%)
Acute rejection (before PJP)	10 (20.4%)
Opportunistic infection (before PJP)	14 (28.6%)
**Comorbidities**	
Pre-transplant diabetes	9 (18.4%)
New onset diabetes after transplantation	3 (6.1%)
Heart failure	5 (10.2%)
Chronic lung disease	4 (8.2%)
Parathyroidectomy (before transplantation)	2 (4.1%)
**Symptoms at the hospital admission**	
Febrile dyspnea	25 (51.0%)
Isolated fever	2 (4.1%)
Non febrile respiratory symptoms	16 (32.7%)
Non febrile alteration of general condition	6 (12.2%)
**Biological presentation at the hospital admission, mean** ± **SD**	
Corrected calcium serum levels (mg/dL)	10.0 ± 1.3
Serum Phosphorous levels (mg/dL)	3.7 ± 0.1
Serum PTH levels (pg/mL)	67.9 ± 20.2
CD4+ T lymphocytes (10^9^/L)	377 ± 298
C-reactive protein (mg/dL)	5.9 ± 4.5
**Microbiologic data**	
*Pneumocystis* identification, n (%)	
*Positive PCR*	49 (100%)
*Microscopic ascus identification and positive PCR*	16 (32.7%)
**PJP Treatment**	
Time to specific anti-infectious treatment initiation (days), median [Q1; Q3]	3 [1; 7]
Treatment duration, mean ± SD (days)	19.0 ± 6.9
TMP-SMX dose, mean ± SD (mg/kg/day)	42.6 ± 21.2
TMP-SMX discontinuation for adverse event, n(%)	10 (20.4)
**Concomitant complications to PJP**	
Hypercalcemia, n (%)	18 (36.7%)
Acute kidney injury, n (%)	37 (75.6%)
Oxygen dependence, n (%)	12 (24.5%)
Transfer to Intensive Care Unit, n (%)	7 (14.3%)

### Prevalence and characterization of hypercalcemia in PJP-patients

**Figure 1:**
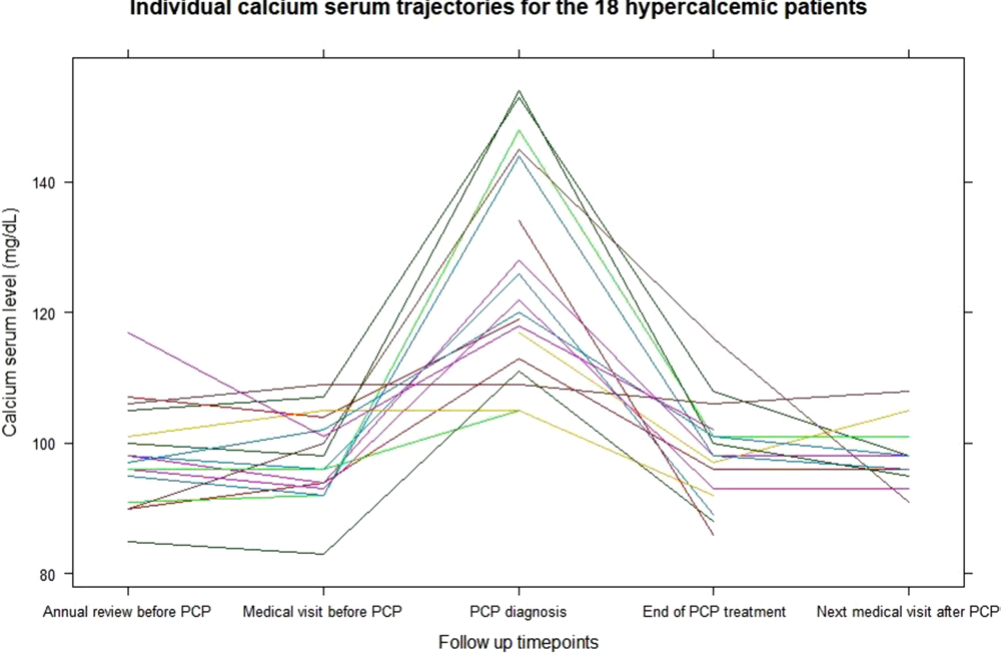
Individual serum calcium kinetic in the 18 hypercalcemic PJP-patients. Representation of serum calcium levels in the peri-infectious period of the hypercalcemic patients. (n = 18), each line representing an individual trajectory/patient. Calcium rates were collected at the. following time points (2 to 5 per patient): at annual consultation preceding PJP (3 to 12 months. before); at the medical visit preceding PJP (2 ± 1 months before PJP); at the time of PJP-diagnosis; at. the end of PJP-treatment; at a medical visit following the PJP resolution (4 ± 2 months after PJP).

Among 49 PJP-patients, 18 (37%) had hypercalcemia at the time of admission (11.9 ± 1.6 *versus* 8.9 ± 1.2 mg/dL in the normocalcemic recipients, *p* < *0.001*). This was concomitant to PJP in all cases; indeed serum calcium values of all patients were normal during the previous clinical evaluation. In the time between admission and PJP-diagnosis, the 18 hypercalcemic patients had a clear elevation of their serum calcium levels (mean calcium peak: 12.6 ± 1.6 mg/dL) that reached more than 14.0 mg/dl in five of them. Three patients required bisphosphonates in order to treat threatening hypercalcemia before PJP-diagnosis was made. In the other patients, hypercalcemia decreased after initiation of specific anti-infectious therapy and serum calcium levels normalized at the end of treatment (from 12.6 ± 1.6 to 9.8 ± 0.8 mg/dL, *p* < *0.001*; Fig. 1).

**Figure 2:**
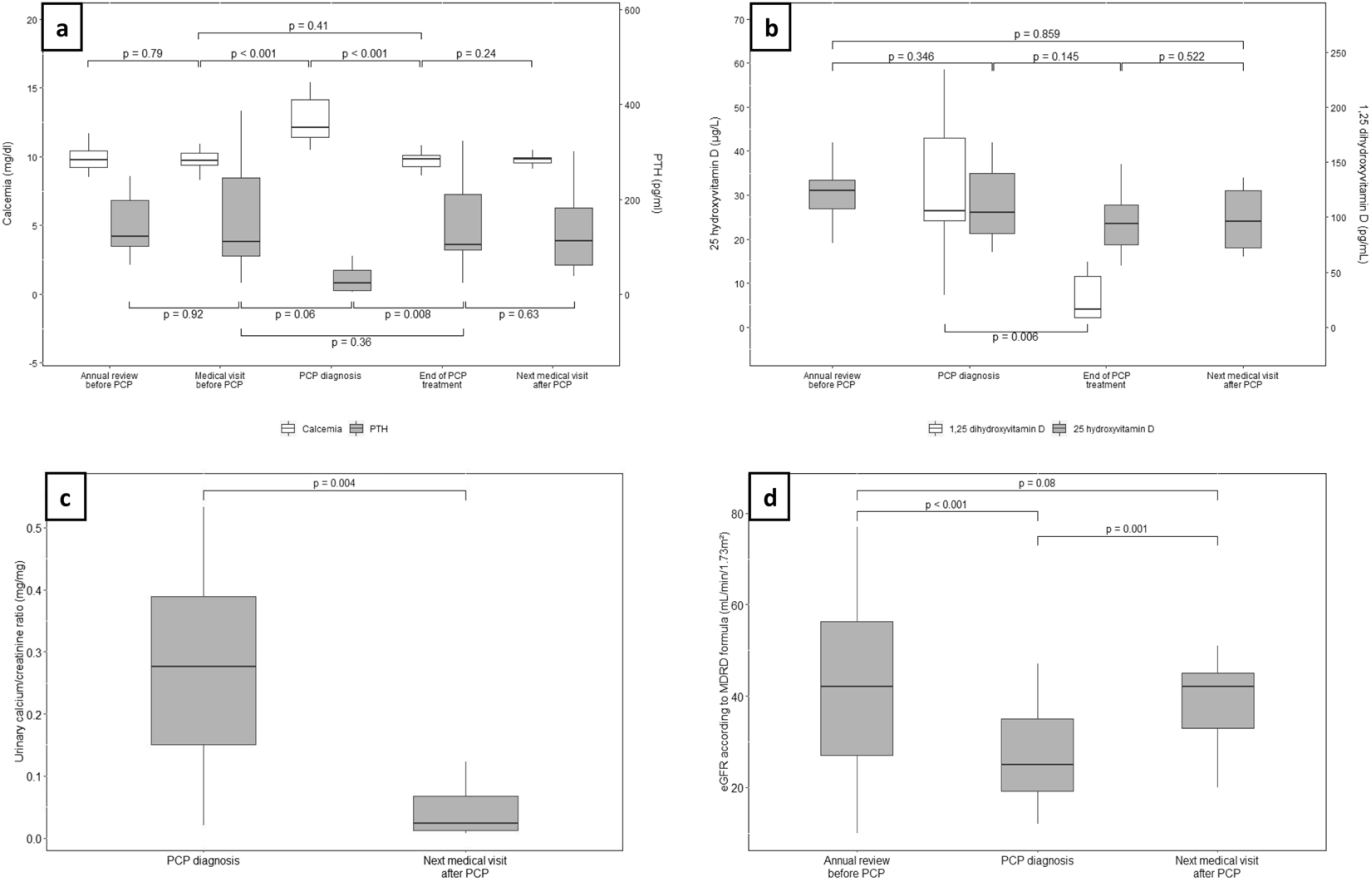
Evolution of mineral metabolism parameters in the 18 hypercalcemic PJP-patients. Representation of mineral metabolism parameters ((**a**) calcium and PTH serum levels; (**b**) 25(OH)D and 1,25(OH)2D serum levels; (**c**) urinary calcium-creatinine ratio; (**d**) estimated glomerular filtration rate by MDRD formula) in the peri-infectious period of hypercalcemic patients (n = 18). The different time points (2 to 5 per patient) correspond to the annual consultation preceding PJP (3 to 12 months before); the medical visit preceding PJP (2 ± 1 months before PJP); the time of PJP-diagnosis; at the end of PJP-treatment; next medical visit following the PJP resolution (4 ± 2months after PJP).

These hypercalcemic-cases were associated with high levels of active 1,25-dihydroxyvitamin D (higher than upper normal value in 10/12 patients with the available data), and significant decrease of PTH serum levels (35 ± 34 pg/mL at PJP-diagnosis *versus* 219 ± 16 pg/mL one to three months before, *p* < *0.001*). After a successful treatment of the PJP episode, we noted a drop in 1,25-dihydroxyvitamin D serum levels (from 124 ± 61.7 to 28 ± 23.2 pg/mL, *p* = *0.006*), and a return of PTH serum levels to their previous values (from 35 ± 34 to 137 ± 99 pg/mL, *p* = *0.009*), as depicted in Fig. 2.

No other obvious cause of hypercalcemia was identified (including primary hyperparathyroidism, multiple myeloma, paraneoplasic hypercalcemia, and native or active vitamin D overdose). There was no significant difference between the two groups of patients, according to the prescriptions of drugs potentially able to interfere with mineral metabolism: there was no difference in immunosuppressive regimen (including CNI and corticosteroids), and none of the patients were prescribed thiazides. In addition, before PJP episode, none of the patients had been treated with calcimimetics, and only 2 required a calcium supplementation, both in the non-hypercalcemic group. No significant difference was noted in the proportion of patients under cholecalciferol supplementation (28 *versus* 29%, *NS*) (Table 2). AKI was common at the time of hospital admission (75.6%) and all patients benefited from a saline hydration protocol in order to manage it.

**Table 2:** Presentation according the normo- or hypercalcemia-associated PJP forms.

	Normocalcemic PJP recipients (n = 31)	Hypercalcemic PJP recipients (n = 18)	*P*-value
Age, mean ± SD	55.8 ± 15.1	52.3 ± 15.6	0.45
Male sex, n (%)	23 (74.2)	13 (73.2)	1.0
Graft function (eGFR, MDRD, ml/min/1.73 m^2^), mean ± SD	39.4 ± 16.8	42.0 ± 18.0	0.62
Mean interval KT-PJP, median [Q1; Q3] (months)	11.4 [6.7; 55.0]	26.5 [10.3; 53.9]	0.44
*Pneumocystis* pneumonia features			
Symptoms at hospital admission, n(%)			**0**.**02**
Febrile dyspnea	20 (64.5)	5 (27.8)	
Isolated fever	1 (3.3)	1 (5.6)	
Non febrile respiratory symptoms	9 (29.0)	7 (38.8)	
Non febrile deterioration in general condition	1 (3.3)	5 (27.8)	
Concomitant complications to PJP			
Acute kidney injury, n(%)	22 (71.0)	15 (83.3)	0.49
Oxygen dependence, n(%)	9 (30.0)	3 (16.7)	0.49
Biological findings at PJP diagnosis, mean ± SD			
CD4 count (10^9^/L)	362 ± 285	404 ± 342	0.74
C-reactive protein (mg/dL)	6.3 ± 5.2	5.0 ± 3.2	0.27
Radiological findings, n(%)			0.95
Apical lesions	5 (17.2)	4 (23.5)	
Basal lesions	7 (24.1)	5 (29.4)	
Diffuse lesions	13 (44.8)	6 (35.3)	
Unilateral lesions	1 (3.5)	0 (0)	
Normal	3 (10.3)	2 (11.8)	
PJP Treatment			
TMP-SMX dose, mean ± SD (mg/kg/day)	44.8 ± 25.1	39.5 ± 14.3	0.41
TMP-SMX discontinuation for adverse event, n(%)	8 (27.6)	2 (12.5)	0.29
Treatment duration, mean ± SD (days)	18.4 ± 8.1	20.2 ± 3.5	0.29
Mineral metabolism *(including interfering drugs)*			
Parathyroidectomy (before transplantation)	2 (6.5)	0 (0.0)	0.53
Calcium supplementation (before PJP), n(%)	2 (6.5)	0 (0.0)	0.53
Cholecalciferol supplementation (before PJP), n(%)	9 (29.0)	5 (27.8)	1.0
Calcimimetic, n(%)	0 (0.0)	0 (0.0)	1.0
Thiazides, n(%)	0 (0.0)	0 (0.0)	1.0
CNI, n(%)	29 (93.5)	16 (94.1)	1.0
Steroids, n(%)	26(83.9)	12 (66.7)	0.29
Main biological findings at PJP diagnosis, mean ± SD			
Serum calcium level (mg/dL)	9.6 ± 0.6	12.6 ± 1.6	<**0**.**001**
Serum albumin level (g/dL)	3.5 ± 0.6	3.4 ± 0.6	0.87
Serum PTH level (pg/mL)	86.6 ± 12.5	34.7 ± 34.0	0.41
25-hydroxyvitamin D level (ng/mL)	14.2 ± 8.6	28.1 ± 8.8	**0**.**008**
1,25-dihydroxyvitamin D (pg/mL)	46.7 ± 44.4	124 ± 61.7	**0**.**06**

### Comparison between hypercalcemic and non-hypercalcemic patients

Comparisons of clinical and biological characteristics of PJP-patients according to their normo- or hypercalcemic status are detailed in Tables 2 and 3.

**Table 3:** Biological parameters according the normo- or hypercalcemia-associated PJP forms.

	Normocalcemic PJP recipients (n = 31)	Hypercalcemic PJP recipients (n = 18)	*P*-value
Serum calcium levels, mean ± SD (mg/dL)			
Annual consultation (3 to 12 months before PJP)	9.1 ± 1.1	9.8 ± 0.8	**0**.**01**
Preceding medical visit (1 to 3 months before PJP)	9.0 ± 1.1	9.8 ± 0.7	**0**.**007**
Day-0 of hospitalization	8.9 ± 1.2	11.9 ± 1.6	**<0**.**001**
PJP diagnosis (calcium serum peak)	9.6 ± 0.6	12.6 ± 1.6	**<0**.**001**
% of serum calcium increase*, median [Q1; Q3]	4.1 [1.1; 8.7]	31.2 [16.2; 43.5]	**<0**.**001**
End of PJP treatment	9.0 ± 0.9	9.8 ± 0.8	**0**.**003**
Next medical visit (2 to 6 months after PJP resolution)	9.1 ± 0.8	9.8 ± 0.5	**0**.**004**
Serum phosphorus levels, mean ± SD (mg/dL)			
Annual consultation (3 to 12 months before PJP)	3.3 ± 0.1	2.9 ± 0.8	0.16
PJP diagnosis	3.6 ± 0.1	3.7 ± 0.1	0.84
Next medical visit (2 to 6 months after PJP resolution)	3.5 ± 0.7	3.5 ± 0.1	0.97
Serum PTH levels, mean ± SD (pg/mL)			
Annual consultation (3 to 12 months before PJP)	194 ± 21.8	179 ± 12.0	0.76
Preceding medical visit (1 to 3 months before PJP)	215 ± 14.2	216 ± 19.0	0.98
PJP diagnosis	86.6 ± 12.5	34.7 ± 34.0	0.41
End of PJP treatment	147 ± 61.8	137 ± 98.9	0.80
Next medical visit (2 to 6 months after PJP resolution)	173 ± 16.1	288 ± 47.2	0.49
Serum 25-hydroxy-vitamin D levels, mean ± SD (ng/mL)			
Annual consultation (3 to 12 months before PJP)	22.4 ± 9.6	30.9 ± 6.9	**0**.**002**
PJP diagnosis	14.2 ± 8.6	28.1 ± 8.8	**0**.**008**
End of PJP treatment	30.3 ± 7.9	24.2 ± 7.7	0.12
Next medical visit (2 to 6 months after PJP resolution)	28.4 ± 10.2	26.9 ± 11.0	0.74
Serum 1,25-hydroxy-vitamin D levels, mean ± SD (pg/mL)			
PJP diagnosis	46.7 ± 44.4	124 ± 61.7	**0**.**06**
End of PJP treatment	46.0 ± 1.4	27.8 ± 23.2	0.15
Graft function			
Annual consultation (3 to 12 months before PJP), eGFR (MDRD, ml/min/1.73 m^2^)	39.4 ± 16.8	42.0 ± 18.0	0.62
Next medical visit (2 to 6 months after PJP), eGFR (MDRD, ml/min/1.73 m^2^)	37.1 ± 17.9	38.6 ± 10.9	0.76
Urine calcium-to-creatinine-ratio, median [Q1; Q3] (mg/mg)			
Annual consultation (3 to 12 months before PJP)	0.03 [0.02; 0.06]	0.02 [0.01; 0.07]	0.67
PJP diagnosis	0.05 [0.02; 0.07]	0.28 [0.16; 0.39]	**0**.**001**

*Serum calcium peak compared to its reference level measured during the previous medical visit (1 to 3 months before PJP).

There was no difference in demographic features (age, gender), immunosuppressive regimen or comorbidities (diabetes, heart failure, chronic respiratory disease). At the time of previous annual transplantation evaluation (3 to 12 months before PJP), serum calcium levels were normal in all patients, but higher in those who would develop a hypercalcemic PJP-form. They also displayed higher 25 hydroxyvitamin D levels than normocalcemic PJP-patients (30.9 ± 6.9 *versus* 22.4 ± 9.6 ng/mL, *p* = *0.002*). The prevalence of pre-transplantation parathyroidectomy, baseline kidney graft function, and phosphocalcic treatments were comparable between the two groups.

Mean time interval between kidney transplantation and PJP was longer in hypercalcemic (26.5 months [10.1–53.9]) than in normocalcemic PJP-patients (11.4 months [6.7–55]) but the difference was not significant. The clinical presentation differed between the two groups. Typical febrile dyspnea was 2-times more common in normocalcemic compared to hypercalcemic patients (66.7 *versus* 27.8%), while an isolated deterioration in general condition (asthenia and/or anorexia and/or weight loss) was noted as the only cause of hospital admission in 28% of hypercalcemic (*versus* 3% in normocalcemic) patients (p = 0.02). Thoracic computed tomography or radiography was performed in 31 and 18 patients, respectively; the radiological presentation of PJP did not differ between the two groups.

At the time of PJP-diagnosis, the increase magnitude in serum calcium level (basal *versus* peak rate) was significantly higher in the hypercalcemic compared to the normocalcemic group (+30 *versus* +4%, p < 0.001). Interestingly, hypercalcemic patients tended to have higher 1.25 dihydroxy-vitamin D (124 ± 61.7 *versus* 46.7 ± 44.4 pg/mL, *p* = *0.06*) and 25-hydroxyvitamin D levels (28.1 ± 8.8 *versus* 14.2 ± 8.6 ng/mL, *p* = *0.008*) as compared to normocalcemic patients. Urinary calcium/creatinine ratio was also higher in hypercalcemic PJP-patients (0.28 [0.16; 0.39] *versus* 0.05 mg/mg [0.02; 0.07], *p* = *0.001*). No significant difference was noted in the proportion of patients under cholecalciferol supplementation (28 *versus* 29%, *NS*), or presenting concomitant AKI (83 *versus* 71%, *NS*).

## Discussion

This work reports a high prevalence of hypercalcemia during PJP in the KTR population, and our results corroborate the hypothesis of an overactive 1-α-hydroxylase supposedly mediated by an infectious granulomatous reaction. Apart from increased levels of 1,25 dihydroxyvitamin D, hypercalcemic PJP-patients also had higher rates of 25 hydroxyvitamin D, and their clinical presentation appeared to be more indolent (less frequent febrile dyspnea). We thus suggest that unexplained hypercalcemia in KTR might reveal a potential underlying PJP, even in the absence of infectious/respiratory symptoms. However, all these hypotheses and conclusions should be taken with caution, due to the retrospective design of the study and its limited number of observations, although this remains the largest series of PJP-associated hypercalcemia.

Few studies have analysed the mineral metabolism in the context of a *Pneumocystis* infection and, to our knowledge, only Hajji *et al*. detailed these elements in their PJP series^18^. This retrospective study included 15 KTR presenting PJP between 2005 and 2007, and reported hypercalcemia in 5 of them (33.3%). At diagnosis mean serum calcium was 11.6 (SD 0.08; range 10.8–12.7) mg/dL, and was associated with decreased rates of PTH and upper normal levels of 1,25 dihydroxyvitamin D. We here report a comparable prevalence of PJP-associated hypercalcemia in our cohort of KTR (37%). It should be noted that ionized calcium levels were not evaluated; nevertheless, the selected cut-off value for hypercalcemia (10.5 mg/dL), and the comparable levels of serum albumin in the two groups argue for a true hypercalcemia. We also observed an adapted PTH level that decreased in all hypercalcemic-patients. In our series, the levels of 1,25 dihydroxyvitamin D exceeded the upper normal value in 83% of hypercalcemic PJP patients (n = 10/12 with the available data), while they were normal in all tested normocalcemic patients. Therefore we hypothesized an extra renal production of 1,25 dihydroxyvitamin D due to a granulomatous reaction against *Pneumocystis*. Indeed, a transient increase of 1,25 dihydroxyvitamin D concentration associated with hypercalcemia has been already described in patients with PJP^13,16,18^. Similar to other infectious or inflammatory granuloma processes (e.g. tuberculosis, cryptococcal disease, sarcoidosis), granulomatous forms of PJP could also lead to an endogenous production of 1 α-hydroxylase *via* activated macrophages^19^, resulting in a raised hydroxylation of 25 hydroxy- to 1,25 dihydroxyvitamin D, increasing both bone resorption and digestive calcium absorption^23^. Supporting the role of PJP in the occurrence of hypercalcemia, mineral metabolism associated parameters also normalized or returned to their basal status after resolution of the infection. Thus, the high frequency of hypercalcemia (more than a third of patients), the chronology between its occurrence/correction and the confirmed diagnosis/resolution of PJP-infections, and the underlying pathophysiological substrate (role of the 1,25 dihydroxyvitamin D) allow us to hypothesize a direct association between hypercalcemia and PJP in KTR.

In order to identify potential specificities of PJP-associated hypercalcemia, we compared the features of hyper- and normo-calcemic patients. The basal levels of serum calcium were higher (although within normal range) in patients who developed PJP-associated hypercalcemia in the year before and after the infectious episode. At the first time point, 25 dihydroxyvitamin D levels were significantly higher in the hypercalcemic-patients, while hyperparathyroidism and chronic graft dysfunction frequencies were similar in both groups.

This increase in 1,25 dihydroxyvitamin D levels might be related to a granulomatous reaction against PJP as discussed above. Conversely 25 hydroxyvitamin D levels were lower in normocalcemic patients (although they were rather low in the hypercalcemic group). Since 1,25 dihydroxyvitamin D formation requires correct rates of 25 hydroxyvitamin D substrate^28^, we hypothesized that the combination of high levels of 1,25 dihydroxyvitamin D and the normal rates of 25 hydroxyvitamin D could have led to hypercalcemia. Since the proportion of patients receiving cholecalciferol supplementation is similar in both groups of our cohort, the difference in 25(OH)D levels could only be related to the individual compliance for vitamin D supplementation, to our opinion. This could also explain why this same difference was also reported several months before PCP-occurrence and why there is no more difference after cessation of cholecalciferol in the hypercalcemic group.

We also found that the clinical presentation corresponded to subacute PJP in hypercalcemic patients who frequently exhibited non-specific deterioration in general condition without fever and dyspnea. These results are consistent with previous descriptions of indolent forms of PJP^11,12,16^, and underline the need to consider atypical manifestations of late PJP in KTR patients.

Interestingly, most of the cases of PJP-associated hypercalcemia reported in the literature occurred in KTR rather than other immunocompromised patients. Indeed, only three cases have been described in acquired immunodeficiency syndrome and bone marrow transplant patients, all before the year 2000^8–10^. In order to explain the susceptibility of kidney transplant recipients to develop hypercalcemia during PJP, one should compare the occurrence of this symptom between this population and another immunocompromised group. It is possible that systematic monitoring of mineral and bone parameters, as usually performed in the KTR population, could lead to more frequent diagnosis of hypercalcemia, especially in the pauci-symptomatic forms. However, some features, specific to KTR, are probably involved: the impairment of urinary excretion of calcium due to chronic graft dysfunction; tertiary hyperparathyroidism, which is common in these patients and limits the negative hormonal feedback on PTH levels; vitamin D supplementation that promotes digestive absorption of calcium.

Although there are now several cases describing PJP-associated hypercalcemia in a context of suppressed PTH and elevated 1,25 dihydroxyvitamin D serum levels, the pathophysiological mechanism involving an 1α-hydroxylase production by a granulomatous reaction remains a hypothesis that has not been demonstrated on a molecular level^19^.

The ability of pulmonary alveolar macrophages to produce 1,25(OH)2D has also been clearly demonstrated in sarcoidosis and in other various granulomatous diseases, such as Wegener’s or silicone-related granulomatosis for example. This has also been described in various infections, such as tuberculosis, histoplasmosis, *Bartonella henselae* infection, and fungal infections^29,30^. However, PJP is not listed as a differential diagnosis for extraparathyroid hypercalcemia. There are few evidences of the presence of a granulomatous reaction directly linked to *Pneumocystis jirovecii*^16,21,22^. Thus, this would be of interest to demonstrate a pathophysiological link between PJP and the secretion of 1,25(OH)2D, by highlighting a potential metabolism of 25 hydroxyvitamin D in cultured pulmonary alveolar macrophages of PJP infected patients, as it has been made for sarcoidosis for example^31,32^.

Based on our findings, and the strong suspicion of an 1,25(OH)2D-mediated mechanism, the use of glucocorticoids should be examined in case of PJP-associated hypercalcemia. In this particular situation, glucocorticoids could be an adequate therapeutic, since the extrarenal 1α-hydroxylase synthesis appears to be suppressible by this molecule^30,33,34^. Moreover, in our cohort, more than 75% of the patients were already receiving daily steroids. For them, we could recommend a temporary increase of the dose, with caution to the potential side effects^35^. Moreover, one should also remind that steroids are already used in case of severe hypoxemic PJP forms, even if their benefits remain controversial^36,37^. As we also addressed it in our work, some of the patients presented with severe hypercalcemia (5/18 with a corrected calcium serum level >14.0 mg/dL), often resistant to the initial medical treatment. In our cohort, three of them received bisphosphonates before the diagnosis has been made, although these molecules are not harmless in a context of impaired renal function^38^. For those three patients, bisphosphonates were fortunately well-tolerated and also efficient, as expected, since these drugs are able to inhibit the potential 1,25(OH)2D-mediated bone resorption^39,40^.

In conclusion, we reported hypercalcemia as being a frequent biological symptom associated with PJP in a KTR population, which taken together with the chronology of the infection process and its resolution, suggests a non-fortuitous association. Moreover, concomitant high 1.25(OH)2D and decrease in PTH serum levels could evoke a granulomatous mediated-mechanism. Consequently, unexplained low-PTH hypercalcemia should lead physicians to suspect a *Pneumocystis* infection in KTR, even in the absence of infectious/respiratory symptoms.
